# Project – Report on the health and social situation of children in Brandenburg

**DOI:** 10.17886/RKI-GBE-2018-058

**Published:** 2018-05-16

**Authors:** Ines Weigelt-Boock

**Affiliations:** Ministry of Labour, Social Affairs, Health and Family of Land Brandenburg

## Overview

Brandenburg’s federal state framework agreement (LRV) on the implementation of the national prevention strategy according to section 20f of Book 5 of the German Social Code specifies the structural and organisational bases for the further development of prevention and health promotion strategies. The targets and fields of action defined by the federal framework recommendations form part of the established structures of health target processes. The Alliance for Growing up Healthy in Brandenburg (BGA) therefore defines health targets with regard to the objective Growing up healthy stated in the federal framework recommendations. LRV partners thereby have agreed to develop activities sustainably and uphold the current quality standards. This includes a commitment to answering the needs revealed by health and social reporting.

In 2018, the roundtable Starke Kinder – Starke Familien (Strong children, strong families) focused on ‘child health and poverty’. This year the thematic focus will be on applying the LRV as a basis for co-operative strategies to improve health equity for children and adolescents at the district and municipal levels.

## Objectives

The planned report on the health and social conditions of children in Brandenburg aims to reveal the causes of health inequality and provide a basis for measures. To evaluate health and social conditions, the report will build on previous reports on child health in Brandenburg. This combined form of health and social reporting references multiple activities in Brandenburg and in addition to the efforts in the context of the Strong children, strong families roundtable, aims to further develop integrated municipal level prevention strategies, as well as implement Germany’s National Prevention Strategy. The report matches health findings to the health targets of the growing up healthy alliance. The Koordinierungsstelle Gesundheitliche Chancengleichheit Brandenburg (coordinating body health equity in Brandenburg) accompanies the establishment and implementation of co-operation projects at the district and municipal level.

## Challenges

Brandenburg’s health and social reporting provides comprehensive data and thereby a basis for monitoring the health and social conditions of children and adolescents. A key challenge remains improving the implementation of these findings in municipal level health planning.

